# [Retracted] Paclitaxel induces apoptosis and reduces proliferation by targeting epidermal growth factor receptor signaling pathway in oral cavity squamous cell carcinoma

**DOI:** 10.3892/ol.2025.14894

**Published:** 2025-01-20

**Authors:** Jing Hu, Na Zhang, Ronglin Wang, Fei Huang, Guang Li

Oncol Lett 10: 2378–2384, 2015; DOI: 10.3892/ol.2015.3499

Following the publication of this paper, it was drawn to the Editor's attention by a concerned reader that the cell apoptotic assay data shown in Fig. 2B on p. 2380 and certain of the western blotting data shown in Fig. 3 on p. 2381 had already appeared in an article written by different authors that had already been submitted for publication.

Owing to the fact that the contentious data in the above article had already been submitted for publication prior to its submission to *Oncology Letters*, the Editor has decided that this paper should be retracted from the Journal. The authors were asked for an explanation to account for these concerns, but the Editorial Office did not receive a reply. The Editor apologizes to the readership for any inconvenience caused.

